# Seeking high-priority mutations enabling successful antibody-breeding: systematic analysis of a mutant that gained over 100-fold enhanced affinity

**DOI:** 10.1038/s41598-020-61529-7

**Published:** 2020-03-16

**Authors:** Hiroyuki Oyama, Yuki Kiguchi, Izumi Morita, Chika Yamamoto, Yuka Higashi, Miku Taguchi, Tatsuya Tagawa, Yuri Enami, Yuriko Takamine, Hanako Hasegawa, Atsuko Takeuchi, Norihiro Kobayashi

**Affiliations:** Kobe Pharmaceutical University, 4-19-1, Motoyama-Kitamachi, Higashinada-ku, Kobe, 658-8558 Japan

**Keywords:** Biotechnology, Immunology, Molecular biology

## Abstract

“Antibody-breeding” has provided therapeutic/diagnostic antibody mutants with greater performance than native antibodies. Typically, random point mutations are introduced into the V_H_ and V_L_ domains of parent antibodies to generate diverse libraries of single-chain Fv fragments (scFvs), from which evolved mutants are selected. We produced an scFv against estradiol-17β with 11 amino acid substitutions and a >100-fold improved affinity constant (*K*_a_ = 1.19 × 10^10^ M^−1^) over the parent scFv, enabling immunoassays with >30-fold higher sensitivity. We systematically analyzed contributions of these substitutions to the affinity enhancement. Comparing various partial scFv revertants based on their *K*_a_s indicated that a revertant with four substitutions (V_H_-L100gQ, V_L_-I29V, -L36M, -S77G) exhibited somewhat higher affinity (*K*_a_ = 1.46 × 10^10^ M^−1^). Finally, the V_H_-L100gQ substitution, occurring in V_H_ complementarity-determining region (CDR) 3, was found to be the highest-priority for improving the affinity, and V_L_-I29V and/or V_L_-L36M cooperated significantly. These findings encouraged us to reconsider the potential of V_H_-CDR3-targeting mutagenesis, which has been frequently attempted. The substitution(s) wherein might enable a “high rate of return” in terms of selecting mutants with dramatically enhanced affinities. The “high risk” of generating a tremendous excess of “junk mutants” can be overcome with the efficient selection systems that we developed.

## Introduction

As exemplified by the fact that pioneers in the field received the Nobel Prize in Chemistry in 2018^1^, the “antibody-breeding” approach (*i.e*., *in vitro* molecular evolution of antibody molecules) revolutionized the generation of therapeutic and diagnostic antibody agents^2–5^. Standard strategies^2–5^ rely upon the introduction of random point mutations or site-directed mutations into the heavy and light chain variable (V_H_ and V_L_) domains of a parent antibody to generate diverse libraries of mutated antibody fragments, *e.g*., single-chain Fv fragments (scFvs)^6,7^ or Fab fragments. Mutated fragments with improved binding characteristics contained therein are then selected and isolated with the aid of genotype-phenotype linking technology^2^, *e.g*., phage display^8,9^, ribosomal display^10^, or yeast cell-surface display^11^.

**Figure 1:**
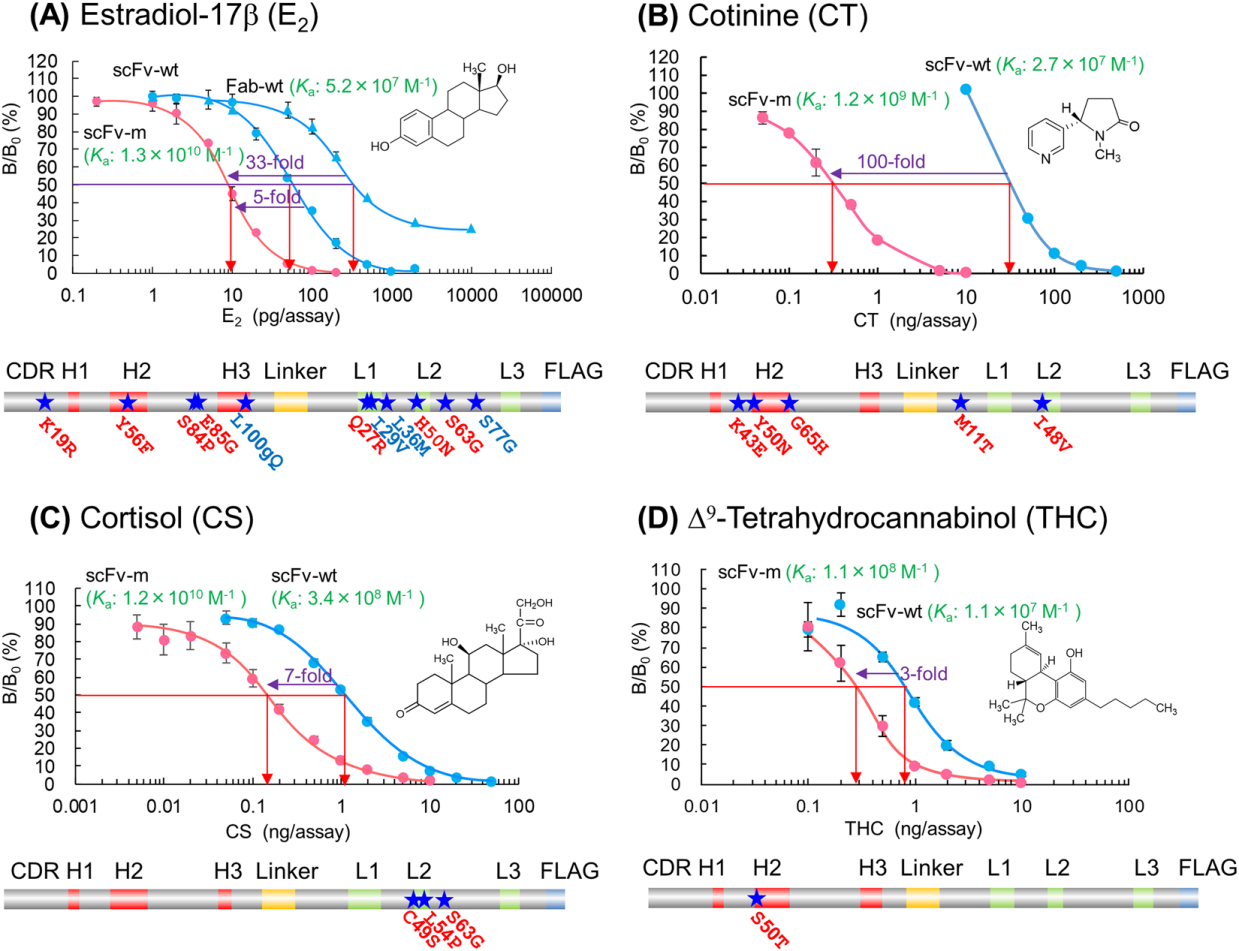
Summary of our previous “antibody-breeding” experiments with scFvs against (**A**) estradiol-17β^12–^^14^, (**B**) cotinine^15^, (**C**) cortisol^16^, and (**D**) Δ^9^-tetrahydrocannabinol^17^. Typical dose–response curves in competitive ELISAs using the wild-type scFv (scFv-wt; blue), wild-type Fab [Fab-wt; blue, shown only in (**A**)] and affinity-matured scFv mutants (scFv-m; magenta) are shown together with the respective *K*_a_ values. These scFv-ms were named in the original articles as (**A**) scFv#m3-*a*18^14^, (**B**) scFv#m1-54^15^, (**C**) scFv#m1-L10^16^, and (**D**) scFv#m1-36^17^. The vertical bars indicate the SDs for intra-assay variances (*n* = 4). The magnitude of improvements in the assay sensitivities (calculated based on the ratios of the midpoint values) are also shown. The primary structure of the scFv-ms, all assembled in the orientation of V_H_–linker–V_L_–FLAG tag, are illustrated. V_H_-CDR1, 2, 3, V_L_-CDR1, 2, and 3 are abbreviated as H1, H2, H3, L1, L2, and L3, respectively. The amino acid substitutions introduced are denoted with dark blue stars and one-letter codes. We should note that, in the original article where the anti-E_2_ scFv#m3-*a*18 (**A**) was generated^14^, we estimated the amino acid at the V_H_-100g position as glutamic acid, based on the behaviors of scFvs in ELISAs. Recently, however, we chemically assigned this residue as glutamine (Q) by LC/MS/MS^49^, as shown in this figure and discussed in this article.

To date, we have produced affinity-matured scFv mutants binding to small biomarkers [*e.g*., estradiol-17β (E_2_)^12–14^, cotinine^15^, cortisol^16^, and Δ^9^-tetrahydrocannabinol (THC)^17^] to establish more sensitive immunochemical assay systems. Actually, these mutants enabled 3–100-fold higher sensitivity in competitive enzyme-linked immunosorbent assays (ELISAs), compared with the corresponding parental scFvs (Fig. 1). Because these scFvs were generated via mutagenesis based on error-prone polymerase chain reaction (PCR) experiments^12–18^, the missense mutations (causing amino acid substitutions) were introduced randomly. Consequently, some of them might function as “key mutations” for increasing in the affinity, whereas other mutations might be “junk mutations” that contribute little, nothing, or even decrease the affinity. Our previous results (Fig. 1) indicated the number of substitutions correlated with the extent of affinity improvement; thus, the anti-THC scFv with a single substitution gained 10-fold higher affinity (based on the calculated equilibrium affinity constant *K*_a_), whereas the anti-E_2_ scFv with 11 substitutions exhibited a *K*_a_ that was increased (improved) by >100-fold. The scFv mutants against cortisol and cotinine showed intermediate improvement, exhibiting >30-fold and >40-fold higher *K*_a_s as the results of three and five substitutions, respectively. However, a reasonable explanation for such correlations requires evidence that most or many (if not all) of these multiple substitutions participated in increasing the affinity to at least some extent.

Regarding the positions of the substitution(s), a remarkable difference was found between the anti-cortisol scFvs (only in the V_L_) and the anti-THC (only in the V_H_) (Fig. 1). In contrast, with the anti-E_2_ and anti-cotinine scFvs that showed more significant improvements, the substitutions were spread over both the V_H_ and V_L_ domains. The most successful example of *in vitro* affinity maturation for an antibody against a small compound was reported for an scFv against fluorescein derivative (*K*_a_ > 1 × 10^12^ M^−1^), which represented an affinity increase of>2,000-fold^19^. This “super” mutant, which is almost beyond native antibodies, was the product of 14 different substitutions, 12 of which were located in the V_H_ domain (Supplementary Fig. S1A).

Recently, some approaches have been developed for improving antibody functions via computational analysis, in order to minimize the trial and error that is an inevitable aspect of the conventional strategies^20–23^. However, using an empirical approach toward more efficient mutagenesis strategies, *e.g*., targeting more limited regions (hotspots) with more controlled amino acid-substitution patterns is still important. To collect useful information, we sought the highest-priority substitutions for successful affinity maturation, among numerous randomly introduced multiple substitutions. We simultaneously pursued the greatest enhancement in affinity that was achievable with the fewest substitutions.

We selected our affinity-matured anti-E_2_ scFv with a 10^10^-order *K*_a_ and 11 substitutions^14^ as the subject of this study. Our novel approach employed here, *i.e*., systematic analysis using partial scFv revertants (scFvs with some substituted amino acids restored to the original sequence), revealed that the most critical substitution among the 11 was the one from leucine (L) to glutamine (Q) at the V_H_100g position, which occurred in complementarity-determining region (CDR) 3 of the V_H_ domain (V_H_-CDR3). This mutation alone resulted in 17-fold enhanced *K*_a_ compared with the wild-type scFv (*i.e*., the parent scFv without any artificial mutations).

## Results

### Origin, and structural and binding characteristics of target anti-E_2_ scFv (scFv#M3rd)

**Figure 2:**
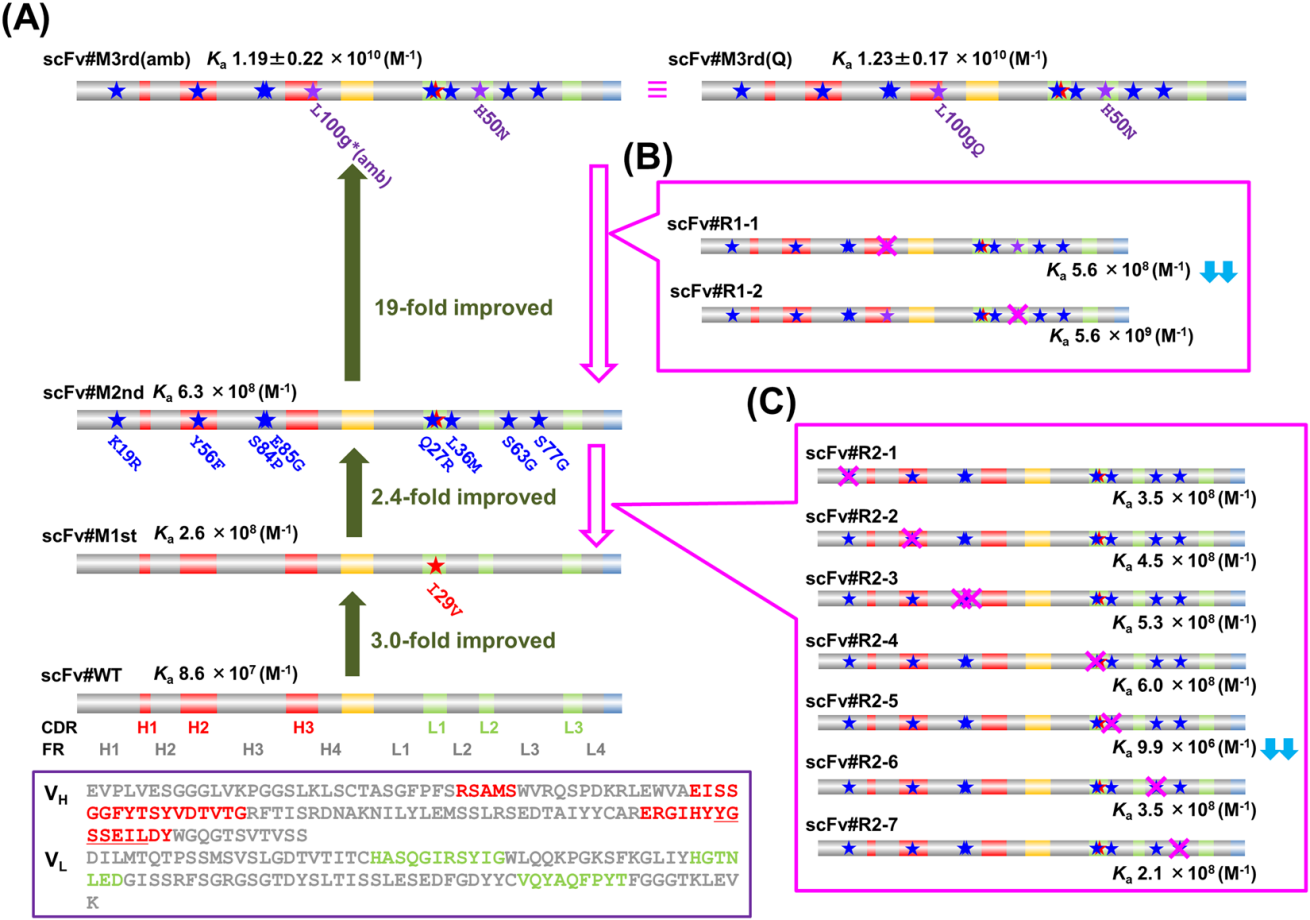
(**A**) Summary of the process used for generating affinity-matured scFvs against estradiol-17β (Ε_2_)^12–14^. Three steps of genetic evolution, *i.e*., scFv#WT (the wild-type scFv combining the V_H_ and V_L_ domains derived from a mouse anti-E_2_ antibody)^12^ → scFv#M1st^12–14^ → scFv#M2nd^13,14^ → scFv#M3rd(amb)^14^, were performed, each of which involved random mutagenesis based on error-prone PCR and phage display-aided selection of improved species. The amino acid sequences of the wild-type V_H_ and V_L_ domains are shown in the purple box. The V_H_- and V_L_-CDRs, determined with the Kabat definition^25^ (H1, H2, H3, L1, L2, and L3), are shown with red and green, respectively. The *K*_a_ values of each scFv, determined by the Scatchard analysis^28^, are shown together with the increasing magnitudes observed with each step. The primary scFv structures are schematically illustrated, where the new amino acid substitution(s) introduced during the first, second, and third mutagenesis steps is represented with a red, dark blue, and purple star(s), respectively. The amino acids before and after each substitution are indicated with the one-letter code. (**B,C**) Schematic illustration of the primary structures of the scFvs introduced with a reverse mutation(s) [(shown with magenta cross(es)] for returning upstream (**B**) from scFv#M3rd(amb) to scFv#M2nd or (**C**) from scFv#M2nd to scFv#M1st. Two substitutions were simultaneously restored in scFv#R2-3. The downward double arrows (↓↓) mean >10-fold decrease in the affinity compared with the parent scFv before reverse mutation(s) was introduced.

The anti-E_2_ scFv focused on in this study, named scFv#M3rd(amb) here [this was originally reported as “scFv#m3-*a*18” (see Fig. 1A)], is an affinity-matured mutant that showed a 10^10^-range *K*_a_ value against free (*i.e*., not immobilized) E_2_ molecules. This scFv was our “third-generation mutant” that was generated previously in our laboratory after three iterative mutagenesis and selection steps performed on the wild-type scFv (scFv#WT)^12–14^ (Fig. 2A). scFv#WT was constructed by linking the V_H_ and V_L_ domains of a mouse anti-E_2_ antibody (Ab#E4-4)^12^ via a common linker sequence composed of glycine (G) and serine (S) in the sequence of (GGGGS)_3_^6,7^ and attaching a FLAG tag^24^ at the C-terminus. The V_H_ and V_L_ domains contained 124 and 107 amino acids (Fig. 2A)^12^, which belonged to subgroups IIID and V^25^, respectively. In this study, we used the numbering and classifications defined by Kabat *et al*.^25^ The V_H_ domain contained 15-residue CDR3, which is substantially longer than the average length of 8.7 residues for V_H_-CDR3 for mouse antibodies against any antigens^26^ or 8.50 residues for mouse antibodies against haptens^27^. Seven amino acids following the residue at position 100 (underlined in Fig. 2A) are defined as the inserted residues in the Kabat-rule, and were named 100a–100 g. The *K*_a_ values against free E_2_ molecules and amino acid substitutions in this mutant are shown together with those of the first- and the second-generation scFv mutants (scFv#M1st and #M2nd, respectively) in Fig. 2A. Previously, we showed that scFv#M3rd(amb) was specific enough and applicable for use with clinical specimens^14^. The 11 amino acid substitutions in scFv#M3rd(amb) are located both in the V_H_ and the V_L_ domains (five and six substitutions, respectively), and both in CDRs and framework regions (FRs) (five and six substitutions, respectively).

In the *scFv#M3rd(amb)* gene variant, a T→A transversion introducing a nonsense mutation from TTG [encoding L] to TAG (amber termination codon) occurred in the V_H_-100g codon, which encodes the residue near the end of V_H_-CDR3. Considering that we used *Escherichia coli* (*E. coli*) XL1-Blue as host cells, which is an *supE* suppressor strain, this amber codon was expected to be readthrough and translated as Q, and this was confirmed by liquid chromatography/tandem mass spectrometry (LC/MS/MS) fingerprinting of the affinity-purified scFv#M3rd(amb) protein (Supplementary Fig. S2). As further confirmation, we modified the *scFv* gene variant replacing the TAG codon at the position with CAG (encoding Q) via oligonucleotide-directed mutagenesis. The product named scFv#M3rd(Q) exhibited almost the same *K*_a_ (1.23 ± 0.17 × 10^10^ M^−1^) [mean ± standard deviation (SD); (*n* = 3)] as that of scFv#M3rd(amb) (*K*_a_ = 1.19 ± 0.22 × 10^10^ M^−1^), as determined by the Scatchard analysis^28^ (Fig. 2A, Supplementary Fig. S3). By performing sodium dodecyl sulfate-polyacrylamide gel electrophoresis (SDS-PAGE) analysis, we observed that both scFvs migrated as a single band at almost the same relative molecular mass (*M*_r_), which was close to the expected *M*_r_ value (27133) (Supplementary Fig. S2). Chemical identity between scFv#M3rd(Q) and #M3rd(amb) was supported by the LC/MS/MS fingerprinting data of the newly generated scFv#M3rd(Q) (Supplementary Fig. S2).

### Determining the substitutions responsible for enhanced affinity (the first stage)

In this study, we employed the “elimination approach” to discover a minimum and essential set of substitutions responsible for the increased E_2_-binding affinities of scFv#M3rd(amb) or (Q) [denoted as scFv#M3rd(amb/Q), hereafter]. We first evaluated the affinity of partial scFv revertants, where one of the multiple substitutions was restored to the original amino acid. The *K*_a_ values were determined by Scatchard analysis^28^ using tritium-labeled E_2_, taking care to avoid inadequate estimations due to any additional structures like “bridges” that link with signal-groups or proteins necessary for immobilization^29,30^.

We first analyzed the third mutagenesis step that generated scFv#M3rd(amb) with a 19-fold increased *K*_a_ as the consequence of only two amino acid substitutions, *i.e*., the V_H_-L100gQ (confirmed as described above) and the histidine (H) to asparagine (N) substitution at the V_L_50 position (Fig. 2A)^14^. The scFv#R1-1 revertant with a single amino acid restoration at the V_H_100g (Q → L) exhibited a 21-fold decreased *K*_a_ (5.6 × 10^8^ M^−1^), which was quite similar to, and rather lower than that of the parent scFv (*i.e*., scFv#M2nd; *K*_a_ = 6.3 × 10^8^ M^−1^) (Fig. 2B). In contrast, scFv#R1-2 with the V_L_50 N → H restoration maintained 47% of the affinity that observed for scFv#M3rd(amb). These results show that the substitution at the V_H_100g was essential whereas the mutation at the V_L_50 was not critical.

Then, we analyzed the second mutagenesis step involving eight amino acid substitutions that converted scFv#M1st into #M2nd, resulting in a 2.4-fold higher *K*_a_^13^. We produced seven revertants (scFv#R2-1–#R2-7), each with a single restoration, except that scFv#R2-3 contained two restorations at the contiguous V_H_84 and V_H_85 positions (Fig. 2C). Considerably lowered affinity was found only for scFv#R2-5 and #R2-7 (*i.e*., 64-fold and 3.0-fold decreases, respectively), indicating that the methionine (M) at the V_L_36 and the G at the V_L_77 were responsible for the higher affinity. We note here that the *K*_a_ of scFv#R2-5 (9.9 × 10^6^ M^−1^) was significantly lower than that of its parent scFv, scFv#M1st (2.6 × 10^8^ M^−1^). This finding suggests a situation where the 10 substitutions excluding V_L_-L36M, found in scFv#M2nd, should have cooperatively reduced the affinity of its parent scFv (*i.e*., scFv#M1st) significantly (26-fold), but that the effect of the single V_L_-L36M substitution on increasing the affinity predominated.

The first mutagenesis step provided scFv#M1st with a 3.0-fold higher *K*_a_ over scFv#WT. This improvement should be due to a isoleucine (I) to valine (V) substitution at the V_L_29 position, which was solely introduced during this step^12,13^. Therefore, this mutation was estimated to be essentially responsible for the increased affinity of the third-generation mutant, which was confirmed later (see below).

### Determining substitutions responsible for enhanced affinity (the second stage)

**Figure 3:**
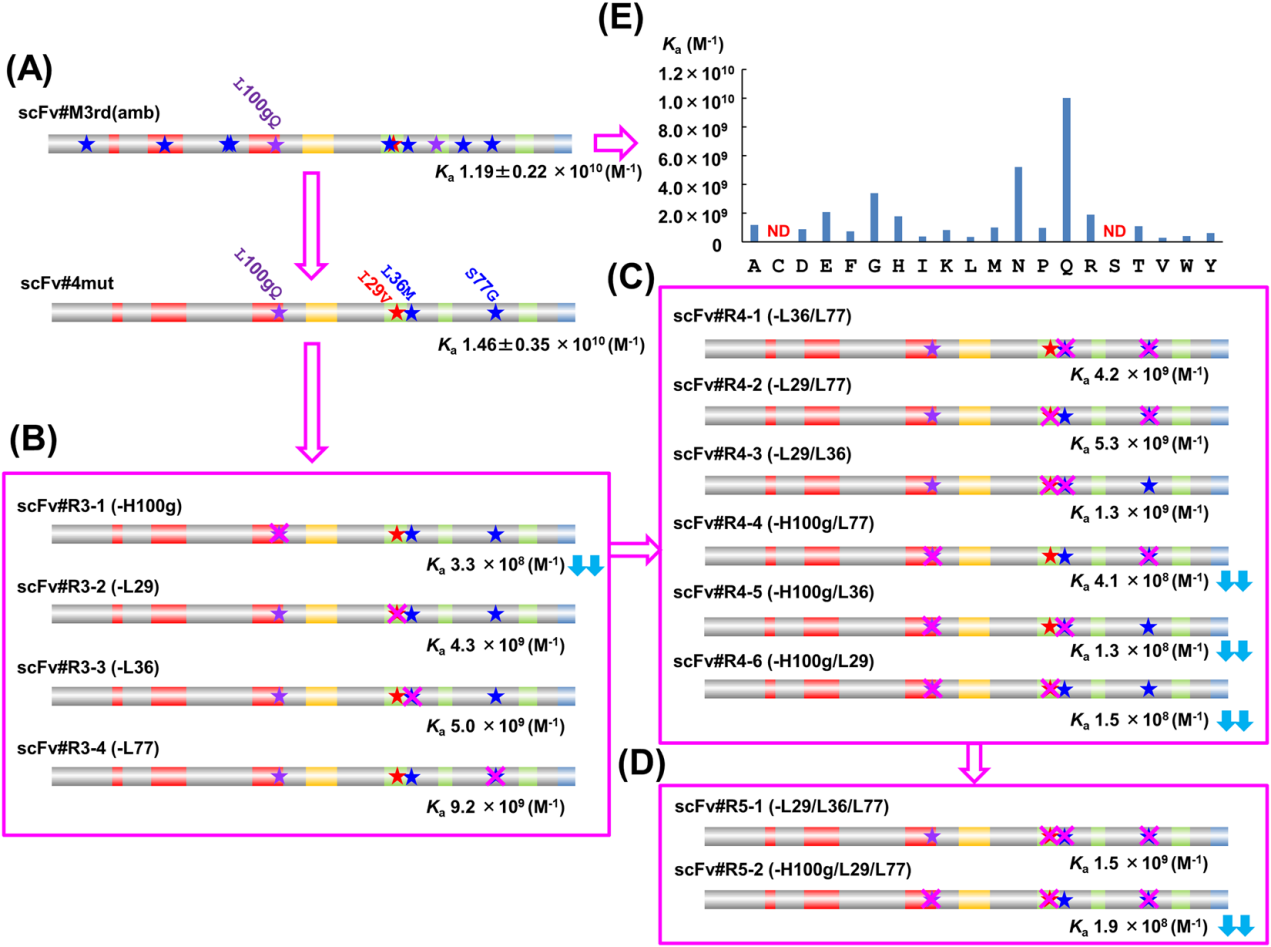
Comparison of the affinities (*K*_a_ values determined by the Scatchard analysis^28^) between (**A**) scFv#M3rd(amb) and scFv#4mut having four substitutions, and between (**B**–**D**) scFv#4mut and its partial revertants retaining (**B**) three substitutions, (**C**) two substitutions, and (**D**) a single substitution. The restored substitutions are shown with magenta crosses. (**E**) The affinities (*K*_a_) of 20 different scFv#M3rd(amb) variants, each of which had a different amino acid at the V_H_100g position (shown in abscissa with the one-letter code), were compared. ND means “not determined” because of too low affinities. The downward double arrows (↓↓) mean >30-fold decrease in the *K*_a_ value compared with scFv#4mut.

During the first-stage examination, we identified the following amino acid substitutions that must have been key for increasing the affinity: V_H_-L100gQ (introduced during the 3rd mutagenesis step), V_L_-L36M and V_L_-S77G (the 2nd mutagenesis step), and V_L_-I29V (the 1st mutagenesis step). However, substitutions selected in different mutagenesis stages do not always function cooperatively. Thus, we generated a new scFv containing these selected four substitutions, named scFv#4mut (Fig. 3A). This mutant showed an even slightly higher *K*_a_ [1.46 ± 0.35 × 10^10^ M^−1^; mean ± SD (*n* = 3)] than that of scFv#M3rd(amb/Q) (*K*_a_ = ~1.2 × 10^10^ M^−1^). Thus, these four substitutions conferred 170-fold greater *K*_a_ over the original scFv#WT, and some of the remaining seven substitutions prevented these desirable four substitutions from enhancing the affinity. To speculate the kinetic mechanism of this affinity enhancement, we compared the association and dissociation rate constants (*k*_a_ and *k*_d_) of scFv#4mut and #WT, determined by the surface plasmon resonance (SPR) sensor. The parameters obtained were as follows: for scFv#4mut, *k*_a_ = 2.97 × 10^4^ M^−1^s^−1^, *k*_d_ = 1.40 × 10^−6^ s^−1^ (*k*_a_/*k*_d_ = *K*_a_ = 2.12 × 10^10^ M^−1^) and for scFv#WT, *k*_a_ = 1.95 × 10^5^ M^−1^s^−1^, *k*_d_ = 6.52 × 10^−3^ s^−1^ (*k*_a_/*k*_d_ = *K*_a_ = 2.99 × 10^7^ M^−1^). These data demonstrate that the improvement in the affinity (706-fold based on the *K*_a_ determined by the SPR) was mainly attributed to the decreased *k*_d_ value of scFv#4mut. In comparison with scFv#M3rd(Q) [*k*_a_ = 1.13 × 10^5^ M^−1^s^−1^, *k*_d_ = 5.05 × 10^−6^ s^−1^ (*k*_a_/*k*_d_ = *K*_a_ = 2.24 × 10^10^ M^−1^)], scFv#4mut is evaluated to exhibit almost similar affinity as a consequence of 3.6-fold less *k*_d_ that compensates for 3.8-fold less *k*_a_.

### Determining substitutions responsible for enhanced affinity (the third stage)

Then, we examined whether even fewer substitutions could cause the increased affinity by analyzing revertants derived from scFv#4mut. Among the four revertants with a single restoration at V_H_100g (Q → L), V_L_29 (V → I), V_L_36 (M → L), or V_L_77 [G → S], named scFv#R3-1, #R3-2, #R3-3, and #R3-4, respectively, scFv#R3-1 exhibited a significantly (44-fold) decreased *K*_a_ compared to scFv#4mut, whereas the decreases of 1.6–3.4-fold were observed with the other three revertants retaining the V_H_-L100gQ substitution (Fig. 3B). These observations strongly suggested that the V_H_-L100gQ substitution was most important for the dramatically increased affinity. Comparison of the *K*_a_ values of scFv#R3-2, #R3-3, and #R3-4 suggested that the extent of cooperation with V_H_-L100gQ might be in the order of V_L_-I29V ≈ V_L_-L36M > V_L_-S77G. This was further analyzed using three revertants with double restorations (Fig. 3C). Regarding the revertants that retained the V_H_-L100gQ substitution, the order of the *K*_a_ was as follows: scFv#R4-2 (containing V_L_-L36M) > #R4-1 (V_L_-I29V) > #R4-3 (V_L_-S77G), all of which showed 10^9^-order *K*_a_ values. However, the other three revertants that lacked the V_H_-L100gQ substitution showed obviously lower affinity (*K*_a_ = 1.3–4.1 × 10^8^ M^−1^).

Finally, we directly evaluated the contribution of the V_H_-L100gQ substitution by determining the *K*_a_ of revertant scFv#R5-1 (Fig. 3D). The result (*K*_a_ = 1.5 × 10^9^ M^−1^) indicated that the single V_H_-L100gQ substitution improved the affinity by 17-fold, suggesting the great potential of performing V_H_-CDR3-directed mutagenesis anew, which has commonly been performed (often with simultaneous V_L_-CDR3-randomization), particularly in early antibody-engineering studies^31–34^. Naturally, we became interested in the potential of substituting the other 18 amino acids (beside Q and the original L) at the V_H_100g position. Thus, we prepared 18 scFv additional mutants by replacing the V_H_100g amino acid of scFv#M3rd(amb) with one of the remaining 18 amino acids, and compared their *K*_a_ values with those of the already-evaluated V_H_-100gQ and V_H_-100gL mutants. As shown in Fig. 3E, the Q-substituted mutant [*i.e*., scFv#M3rd(Q)] exhibited the most improved affinity, and the next highest was the mutant substituted with N (~14-fold improvement over the V_H_-100gL mutant), a homolog of Q with an amide group but with one less carbon atom in the side chain. Moderate, but significant (>5-fold) improvements were observed for the G-, glutamic acid (E)-, H-, and arginine (R)-substituted mutants. Amino acids with aliphatic and hydrophobic side chains (I and V) or aromatic rings [phenylalanine (F), tryptophan (W), and tyrosine (Y)] contributed little to the improvement. It was surprising that substitution with S, though well-recognized as a residue (as well as Y) that often plays important roles in interactions with antigens^35^, deteriorated the affinity down to undetectable range, as also seen with the cysteine (C)-substituted mutant. We should also note that the Q substitution, which increases the binding affinity most, is not among the top 10 amino acids that frequently appear in the CDR sequences of mouse antibodies against haptens [*i.e*., Y, S, G, L, N, W, threonine (T), I, alanine (A), and R]^27^: this suggests that it may be difficult to use a prediction-based approach for improving amino acid sequences in this most diverse CDR^25–27,36,37^. Nonetheless, these data (Fig. 3E) suggested to us the possibility that every position in V_H_-CDR3 might be substitutable with much more potent amino acids that cause dramatically enhanced affinity, but that it should rarely be achieved via error-prone-PCR-based mutagenesis.

### Summary of affinity-maturation results from scFv#WT to scFv#M3rd(amb/Q)

**Figure 4:**
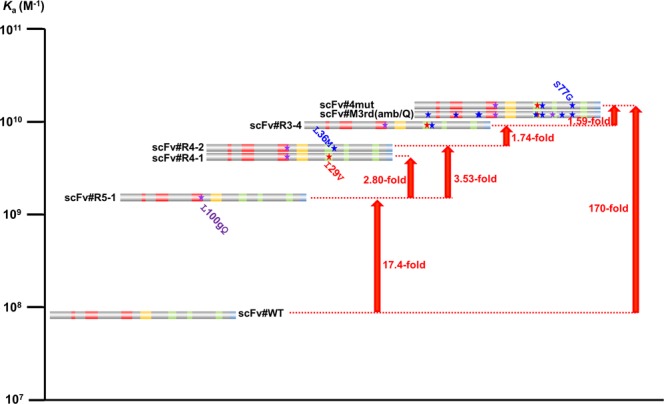
Schematic illustration of the hierarchy of the anti-E_2_ scFvs in terms of the antigen-binding affinity (*K*_a_ determined by the Scatchard analysis^28^). The upward orange arrows indicate increases in the *K*_a_, and the magnitudes are shown beside the arrows.

These findings enabled us to order the affinity-maturation process by focusing the importance of V_H_-L100gQ substitution, as shown in Fig. 4. The most improved mutant species with single, double, and triple substitutions were estimated to be scFv#R5-1, #R4-2, and #R3-4, respectively, the *K*_a_ values of which increased as the numbers of substitutions increased. Comparison of the *K*_a_ values between scFv#R3-1 and R3-3 (Fig. 3B), and between scFv#R4-3 and R4-6 (Fig. 3C), indicates a greater potential of substitution at V_H_-L100gQ than V_L_-L36M under the presence of one or two other substitution(s). These observations were compatible with the result where scFv#R5-2 with a single V_L_-L36M substitution exerted the *K*_a_ value that was only 2.2-fold greater than scFv#WT and much lower than scFv#R5-1. Quadruple substitutions in scFv#4mut were necessary to reach (and even exceed) the affinity of scFv#M3rd(amb/Q), the high-affinity mutant with 11-amino acid substitution. Therefore, assistance of V_L_-S77G substitution was necessary, although it was not as potent as that of V_L_-I29V and V_L_-L36M substitutions. After all, it was shown that, in scFv#4mut, the 4 substitutions functioned additively and showed a 170-fold higher affinity than that of scFv#WT.

### Analytical utility and structural aspects of the high-affinity scFvs

**Figure 5:**
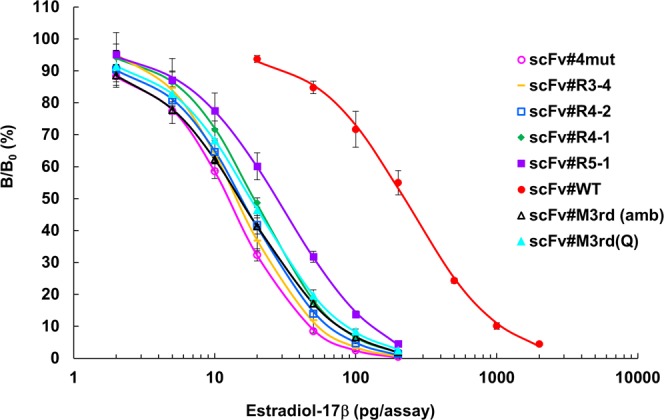
Dose–response curves of competitive ELISAs obtained with the scFvs shown in Fig. 4. The vertical bars indicate SD for intra-assay variance (*n* = 4). The midpoints of the curves (pg/assay) were as follows: scFv#4mut, 12.6 ± 0.12 [mean ± SD (*n* = 4)]; #M3rd(amb), 15.0 ± 0.76; #M3rd(Q), 16.8 ± 1.57; #R3-4, 14.3; #R4-2, 15.6; #R4-1, 19.0; #R5-1, 27.6; and #WT, 220 (average of determinations in duplicate). In these assays, the scFv concentrations were adjusted to give bound enzyme activities at B_0_ (the reaction without E_2_ standard) of approximately 1.0–1.5 absorbance after a 30-min enzyme reaction. The background absorbance (observed without addition of scFvs) was lower than 5.0% of the B_0_ absorbance. We should note that, in the original article where scFv#M3rd(amb) (denoted as scFv#m3-*a*18 therein) was generated^14^, we reported its *K*_a_ value as 1.3 × 10^10^ M^−1^, and the midpoint value in the ELISA using this scFv was determined to be 10.0 ± 1.2 pg/assay. In this study, we re-determined the *K*_a_ in triplicate. The midpoint values were also re-determined to perform equal and strict comparisons between the scFvs, because we had to use a newly prepared E_2_–BSA conjugate to coat the ELISA microplates. Difference in the quality of these conjugates (mainly in the hapten/protein molar ratio) influences on the ELISA sensitivity and often makes it difficult to strictly reproduce previous experimental data.

Immunoassay sensitivities basically correlate with the affinities of the antibodies used, and usually antibodies with a higher affinity enable immunoassays with higher sensitivity^38^. Indeed, scFv#M3rd(amb), #M3rd(Q), and #4mut (showing *K*_a_ values in the 10^10^-range), as well as scFv#R3-4, #R4-1, #R4-2, and #R5-1 (showing *K*_a_ values in the 10^9^-range), displayed dramatically enhanced sensitivities in competitive ELISAs, as shown by 8.0–17-fold lower midpoint values (~13–28 pg/assay) than scFv#WT (220 pg/assay) in dose–response curves (Fig. 5). These dose–response curves with improved sensitivities cover a measurable range required for clinical applications^14^.

**Figure 6:**
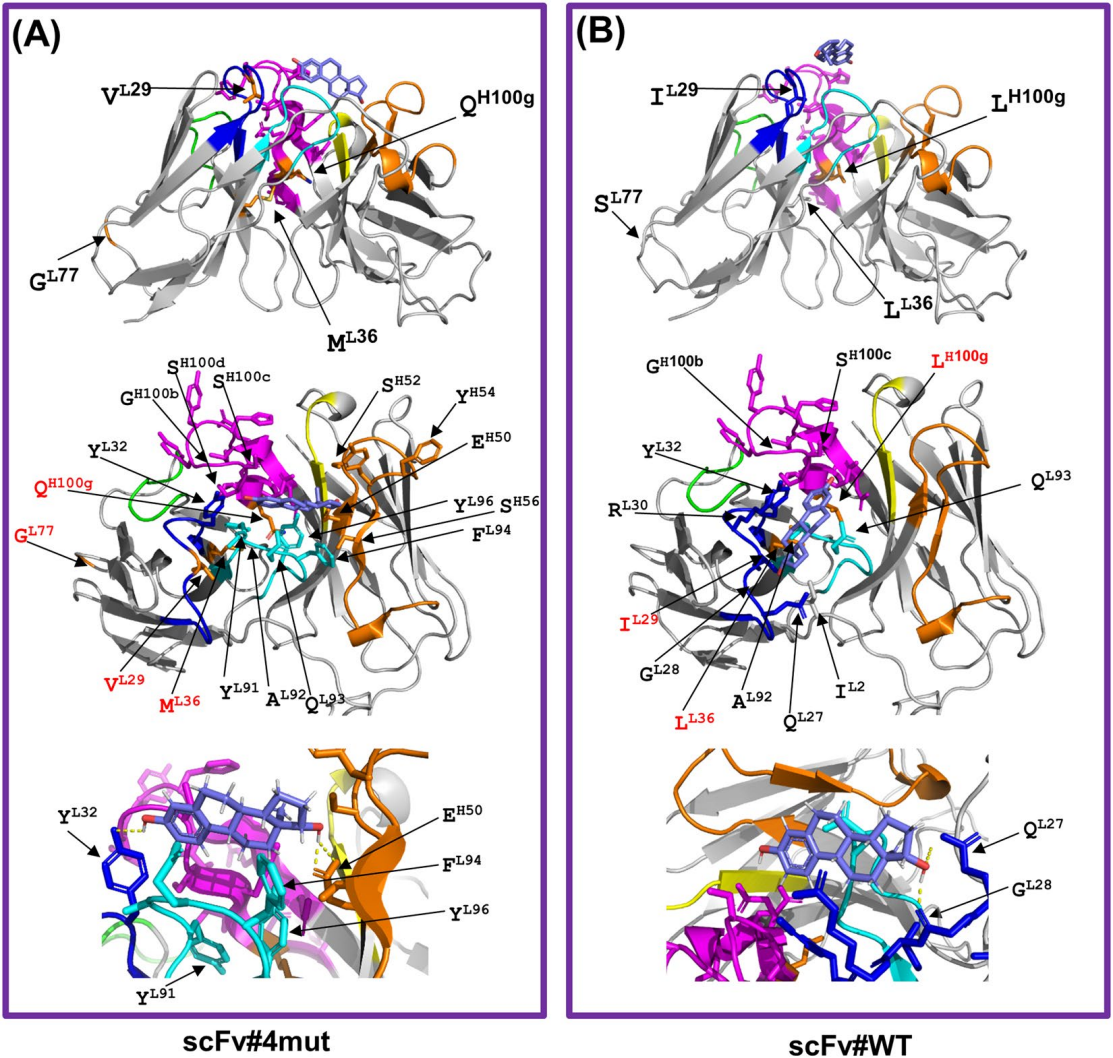
Protein ribbon structures for (**A**) scFv#4mut and (**B**) scFv#WT were constructed using the SWISS-MODEL Protein Modelling Server^50^, and their conformations when docked to E_2_ were predicted using SwissDock^51^. Three different views observed from different angles are shown. In the ribbon representation of the scFv backbones, CDR H1 (yellow), H2 (orange), H3 (magenta), L1 (dark blue), L2 (light green), and L3 (light blue) are represented with β-sheet structures (bold gray arrows). The introduced amino acids after the substitutions are shown in orange. The backbone of the E_2_ molecule is shown in light purple. Image generated with PyMOL^52^.

Protein modeling of the scFv#4mut and #WT, docked with E_2_, is shown in Fig. 6. Although such *in silico* approaches offer structural information with more or less limited reliability compared with X-ray crystallography, the modeling of scFv#4mut strongly suggested that none of the 4 substituted residues (Q at the V_H_100g position, or V, M, and G at the V_L_29, 36, and 77 positions, respectively) forms direct contacts with the E_2_ molecule in the immune complex. The Q residue was located near the *C*-terminus of V_H_-CDR3 and should function by raising the loop structure of this CDR. Substitution from the original L to Q is estimated to alter the steric conformation of V_H_-CDR3 loop, but the modeling did not suggest obvious interaction of V_H_-CDR3-related residues with E_2_ molecule. Instead, a possibility  was shown where, in scFv#4mut, V_H_-CDR2 and V_L_-CDR1 might interact with E_2_. Thus, the hydroxy group of Y residue (at the V_L_32 in the CDR1) and the carboxy group of E residue (at the V_H_50 in the CDR2) might anchor E_2_ molecule via the hydrogen bonds with the hydroxy groups of the A- and D-ring of the steroid skeleton, respectively, resulting in drastic change of the orientation of the E_2_ molecule in the paratope compared with that in scFv#WT. The V (at the V_L_29) and/or M (at the V_L_36) residue(s) introduced in scFv#4mut might trigger such V_L_-CDR-dependent events.

We should note here that both the introduced M residue and original L residue are unusual amino acids as for the V_L_36 position: in fact, the residues at the V_L_35–41, which form the beginning of V_L_-FR2, are highly conserved in the sequence WYQQK(lysine)P(proline)G found in all V_L_ subgroups^25^. The G residue at the V_L_77 position is a member of FR3: the original amino acid was S, which frequently appears at this position. The contribution of this G was not significant as that of the V or M residue, but was essential for increasing the affinity to that observed with scFv#3rd(amb/Q). The mechanism whereby the G residue contributed to the affinity, despite its considerably long-distance from the paratope, is of great interest.

## Discussion

For those of us, working in the fields of analytical and diagnostic chemistry, antibodies with high affinity for target molecules are an essential tool. Because the higher affinity enables more sensitive analytical/diagnostic systems, “antibody-breeding” that generates mutant antibody fragments with improved affinity is an attractive methodology. However, the conventional approach combining random mutagenesis and panning-based selection has very often failed to provide satisfactorily improved mutants, despite much time consuming effort. To overcome such challenges, revolutionary strategies are required for efficiently introducing functional mutations without unnecessarily enlarging the diversity and for reliably selecting rare and improved mutants without overlooking them.

To achieve the former requirement, information is needed for designing “decisive mutations (substitutions)” essential for elevating the antigen-binding affinity. It would also be of great help if it were possible to discover “hot spots” in the V_H_ and/or V_L_ domains of antibodies, where a wide range of mutations introduced thereto significantly improves the affinity. Of course, it is more desirable if fewer mutations introduced in a narrower region facilitate isolating improved species with high probability.

Previously, we summarized the results of studies wherein antibody mutants were generated that bound to small molecules (haptens)^14,38^. Among the affinity-matured products reported, mutant scFvs or Fabs that showed *K*_a_ values greater than 1 × 10^9^ M^−1^ (*i.e*., a standard value required for subpicomole-order analysis), owing to>10-fold enhancement, were selected and their structures are illustrated in Supplementary Fig. S1A. Four out of the six mutants generated, including the scFv with a *K*_a_ value greater than 1 × 10^12^ M^−1^ and the greatest improvement^19^, involved totally 10 or more substitutions and some of them were in V_H_-CDR3, similar to our scFv#M3rd(amb). Because two of these four mutants (*i.e*., anti-fluorescein-biotin and anti-fluorescein antibody fragments) were generated via error-prone PCR^18^ or related methods, only some of the multiple substitutions introduced might have driven the enhanced affinity. Seeking substitutions that do in fact contribute to the binding affinity is inevitable for developing simpler strategies that depend less on trial and error.

Thus, we analyzed our anti-E_2_ scFv#M3rd(amb): this mutant had 11 amino acid substitutions and showed an extremely high (10^10^-order) *K*_a_ against free E_2_ molecules, which was over 100-fold higher than that of the parent antibody (scFv#WT). To explore the hierarchy of these substitutions regarding contribution to the enhanced affinity, we employed a unique approach based on the comparison of the affinities of various partial scFv revertants. Although this case study was performed with a particular antibody mutant, we obtained several suggestive results that exceeded our initial expectations, as summarized below. First, a mutant with only four substitutions showed even higher affinity than scFv#M3rd(amb), *i.e*., the *K*_a_ was 1.46 × 10^10^ M^−1^, corresponding to a 170-fold improvement compared to scFv#WT. The incorporated amino acids (and positions) were Q (V_H_100g), V (V_L_29), M (V_L_36), and G (V_L_77). Second, the most influential residue was Q at V_H_100g (locates in V_H_-CDR3), which alone improved the affinity by 17-fold. Third, none of the substitutions at V_H_100g with the remaining 19 different amino acids resulted in an equivalently improved affinity. Fourth, the extent that these substitutions contributed to the enhanced affinity was in the order of Q ≫ M ≥ V ≫ G, and these four substitutions seemed to function in an additive manner. Consequently, the mutant with three substitutions (Q/M/V residues) showed 63% of the affinity observed with the mutant with four substitutions, and the mutant with two substitutions, Q/M or Q/V, exhibited 36% or 29% affinity, respectively. These mutants with Q/M/V, Q/M, and Q/V substitutions demonstrated 107-fold, 62-fold, and 49-fold higher affinity than the parent scFv#WT, maintaining 10^9^-order *K*_a_ value.

These findings impressed upon us the importance of identifying a decisive substitution (“ace” substitution) like the V_H_-L100gQ substitution described above. Considering the mutation patterns found in other reported high-affinity mutants as well (Supplementary Fig. S1A), the V_H_-CDR3 should be focused on as a “hot region” where such an ace substitution might be discovered with high probability. V_H_-CDR3 often works as the “ace CDR” in antigen recognition^36,37^, and thus at the dawn of antibody engineering (~1990), “hard randomization”^39^ was frequently performed for multiple amino acid residues therein (often together with V_L_-CDR3) by the site-directed introduction of degenerated NNS [*i.e*., (A/C/G/T)(A/C/G/T)(C/G)] or NNK [*i.e*., (A/C/G/T)(A/C/G/T)(G/T)] codons encoding any of 20 different amino acids^32,33^. This was actually a potent strategy for generating new prototype antibody fragments that gained different specificities, but was unlikely to be suitable for improving the affinity while maintaining the original specificity. In fact, our previous affinity-maturated scFvs against cotinine, cortisol, and THC did not contain substitutions in V_H_-CDR3 (Fig. 1B–D).

Such outcomes should be mainly attributable to the highly diversified structure and nature of V_H_-CDR3, each with different binding specificity and affinity function to allow for “the best fitting” against only a limited antigen structures. Therefore, the “hardly” mutagenized libraries only rarely generate satisfactorily improved mutants, which, furthermore, should be buried by tremendously large excess of mutants with deteriorated binding performance. The most popular selection systems, *i.e*., those combining phage display and panning do not always facilitate successful isolation of such rare and hidden species, mainly due to the biased propagation of phage clones displaying antibody fragments and the competition with a large excess of the phage clones displaying antibodies with weaker or deteriorated affinities^40^. Moreover, the hard mutagenesis of more than seven amino acid residues, which should produce >20^7^ (= 1.28 × 10^9^) different amino acid sequences, generates libraries too large to deal with using standard experimental conditions. It is no wonder that these difficulties made us gradually avoid the V_H_-CDR3-focused strategies for the purpose of affinity-maturation.

However, the present findings suggest a much simpler strategy for mining a possible ace mutation(s) in V_H_-CDR3. The parent scFv (scFv#WT) has a V_H_-CDR3 composed of 15 amino acids (Fig. 2A), which also correspond to the definition by Chothia *et al*.^41,42^. We here assumed that 13 of these residues, avoiding the less-variable D and Y residues at the V_H_101 and V_H_102 positions, are important for generating affinity against E_2_. Searching a very small scFv library, *i.e*., a sum of 13 groups of sequences each containing 20 different sequences generated by the hard randomization of one of the 13 positions (therefore, theoretically composed of only 13 × 20 = 260 amino acid sequences), should have enabled discovery of the mutant with the ace V_H_-L100gQ substitution. Even in the cases where two (or more) ace-equivalent mutations were present in the CDR3 and cooperated together, each of them could be separately discovered. Parallel examinations following hard mutagenesis of two or three serial residues (each generates 12 × 20^2^ = 4,800 or 11 × 20^3^ = 88,000 sequences, respectively) might help in finding “ace-mutation motifs”. In our laboratory, development of a novel and efficient strategy for discovering affinity-matured scFv mutants, named “colony-array profiling”, is now progressing. We believe that this approach should be particularly suitable for examining such small libraries and will be of great help, the results of which will be reported in the near future. After finding such an ace mutation, some cooperating mutations (*e.g*., corresponding to the V and M residues in this study) should be searched for. Error-prone PCR might be still suitable for this purpose.

One of the motives for undertaking this study was an article published in 1990, where the author sought amino acid substitutions that caused a >200-fold difference in the *K*_a_ (determined by the fluorescence quenching method) between two hybridoma-derived antibodies against *p*-azophenylarsonate^43^. Although 19 amino acid differences were originally observed between these antibodies, the author finally showed that only three amino acid substitutions (two in V_H_-CDR2, one in V_H_-CDR3) were needed to produce this increased affinity by examining artificial antibodies produced with the aid of synthetic oligonucleotides (Supplementary Fig. S1B). A more striking example was shown by the affinities observed for anti-digoxin antibodies that were separately obtained from different hybridoma clones. Thus, the antibody named 26-10 exhibited 279-fold higher affinity (10^10^-range *K*_a_ as determined by the equilibrium saturation method) than that of the antibody named LB4, surprisingly due to only a single substitution at the V_H_52 position located in V_H_-CDR2^44^ (Supplementary Fig. S1B). In both cases, substitutions in V_H_-CDR2 must have resulted in the dramatically enhanced affinity (with high probability for the former case, and absolutely for the latter case). These results are reasonable because, for antibodies against haptens, V_H_-CDR2 and V_L_-CDR1 tend to play important roles in forming the antigen-binding cavity^45^, and this speculation was supported by the successful affinity maturation of anti-tacrolimus scFvs due to randomizing V_H_-CDR2 and V_L_-CDR1 with NNS codons^46^. However, even in such cases, the approach for seeking ace substitutions mentioned above can easily applied by extending it to more than one CDR in a parallel manner.

We are greatly interested in identifying the greatest possible increase in the affinity with the fewest substitutions, because this suggests the potential of *in vitro* affinity maturation of antibodies. In this study, we achieved 170-fold higher affinity by introducing four substitutions (with scFv#4mut), 107-fold higher affinity by introducing three substitutions (with scFv#R3-4), and 62-fold higher affinity by two substitutions (with scFv#R4-2), and these improved scFv mutants showed >10^9^-order *K*_a_ values. Considering the difficulty in performing extensive improvement from already-matured antibodies (*e.g*., whose *K*_a_ values exceed 10^8^ M^−1^), our present results might be worthy of attention.

## Materials and Methods

### Buffers

The following buffers^12–17^ were used in this study. PB: 50 mM sodium phosphate buffer (pH 7.3); PBS: PB containing 9.0 g/L NaCl; G-PBS: PBS containing 1.0 g/L gelatin; T-PBS: PBS containing 0.050% (v/v) Tween 20; and PVG-PBS: G-PBS containing 1.0 g/L polyvinyl alcohol with an average polymerization degree of 500.

### scFvs

Anti-E_2_ scFv#WT, scFv#M1st, scFv#M2nd, and scFv#M3rd(amb) (originally named as scFv#E4-4^12–14^, scFv#m1-e7^12–14^, scFv#m2-c4^13,14^, and scFv#m3-*a*18^14^, respectively) were prepared as soluble proteins as we described previously^12–14^. Other scFvs having reverse mutation(s) (*i.e*., revertants) were produced by expressing the corresponding *scFv* genes in *E.coli* XL1-Blue cells as described previously^12–17^. These *scFv* genes were constructed by PCR using synthetic oligo-DNAs predesigned to introduce the targeted mutation(s) as shown below, whose nucleotide sequences were confirmed by the standard method. The scFv proteins were obtained as periplasmic extracts from mass-cultured transformants^12–17^, and used for *K*_a_ determinations and ELISAs. For SDS-PAGE analysis, the scFvs were affinity-purified with anti-FLAG M2 antibody-immobilized agarose gel (Sigma–Aldrich)^14-16^.

### Preparation of gene fragments encoding the scFv revertants

Among the 21 *scFv* genes synthesized in this study, typical instances were selected and their preparations are shown below. PCR experiments were performed using an adequate *scFv* gene (subcloned into the pEXmide 5 vector^47^; 0.5–50 ng) as the template in a buffer (100 μL) containing *Ex Taq* (TaKaRa-Bio) (0.5 or 2.5 U) or *KOD Fx* DNA polymerase (TOYOBO) (2.5 U), 20 nmol of each dNTP, and a combination of reverse/forward primers (50–100 pmol each), unless otherwise specified. Usually, the following cycling condition was used: 94 °C(2 min); then 35 cycles of 98 °C for 10 sec, 55 °C for 30 sec, and 72 °C for 1 min, followed by a hold step at 72  °C for 10 min. The nucleotide sequences of the primers are shown in Supplementary Table S1. Every *scFv* gene fragment synthesized was digested with *Nco* I and *Sal* I, ligated with the similarly digested pEXmide 5 vector^47^, and introduced in the *E. coli* cells by electroporation^12–17^.

- **scFv#R1-1**. Three sets of PCRs were performed using the *scFv#M3rd(amb)* gene^14^ as the template and one of the following three combinations of primers: (i) R1 and F1, (ii) R2 and F2, or (iii) R3 and E_2_-V_L_-For-2^12^ (Supplementary Figure S4A). Using the three kinds of PCR products (i–iii), two overlap-extension PCR steps were performed. First, the products i and ii (each 200 ng) were mixed and subjected to 10 cycles of PCR in a 25-μL buffer solution containing *ExTaq* polymerase. A portion of the reaction solution (10 μL) was mixed with R1 and F2 primers and re-amplified similarly, but for 15 cycles in a 100-μL buffer solution containing *ExTaq* polymerase. Second, the resulting product was gel-purified, and a portion (200 ng) was mixed with product iii (200 ng) and subjected to a similar serial two-step amplification to generate the desired gene fragment.
- **scFv#R2-1**. PCR was performed using the *scFv#M2nd* gene^13^ as template with R1 and F3 primers. The product obtained was used as reverse mega-primer (MP) in the next PCR, in combination with the E_2_-V_L_-For-2 primer^12^, to generate the desired gene fragment.
- **scFv#4mut**. PCRs were performed using the *scFv#WT* gene^12,13^ as template (Supplementary Figure S4B). The *V*_*H*_-portion gene was prepared by amplification with E_2_-V_H_-Rev and F4 primers. The *V*_*L*_-portion gene was prepared as follows. First, PCR was performed using R5 and F5 primers. The product obtained was gel-purified and used as reverse MP1 in the next PCR, in combination with the E_2_-V_L_-For-2 primer. The product was then used similarly as forward MP2 with the E_2_-V_L_-Rev primer^12^ to generate the *V*_*L*_-portion gene fragment with 5′-end sequence that was complementary to the 3′-end sequence of the *V*_*H*_-portion gene fragment. These *V*_*H*_- and *V*_*L*_-portion gene fragments (each 200 ng) were mixed and submitted to overlap-extension PCR as described above (see entry **a**) to generate the desired gene fragment.
- **scFv#R3-1**. PCRs were performed using the *scFv#WT* gene as template. The *V*_*L*_-portion gene was prepared as follows. First, PCR was performed using R4 and MP1 primers. The product obtained was gel-purified and used as MP3, which was submitted to overlap-extension PCR with MP2 as described above (see entry **a**) to generate the gene fragment containing whole the *V*_*L*_ with a portion of the 3′-side of *V*_*H*_ (extending over V_H_-CDR3). On the other hand, a gene fragment containing the *V*_*H*_-portion was amplified using E_2_-V_H_-Rev and E_2_-V_H_-For primers. These two gene fragments were combined by the overlap-extension PCR to generate the desired gene fragment.
- **scFv#R4-3**. PCR was performed using the *scFv#WT* gene as template with R2 and F5 primers. The product obtained was digested with *BamH* I and *Sal* I to generate a gene fragment covering the *V*_*L*_ with the mutation that substitutes the V_L_77 residue, whereas the plasmid having *scFv#4mut* gene was digested with *BamH* I and *Sal* I and gel-purified to remove the corresponding gene fragment. The V_L_77-mutated gene fragment was ligated into the digested plasmid to construct the desirable gene as the form already incorporated in the plasmid.
- **scFv#R5-1**. The desired gene fragment was generated by PCR using the *scFv#WT* gene as template with an MP (the *V*_*H*_-portion gene fragment prepared in entry **c**) and the E_2_-V_L_-For-2 primer.

### Determination of the scFv *K*_a_ values

- **Scatchard analysis**^28^. Mixtures of [1, 2, 6, 7-^3^H]-E_2_ (3.53 TBq/mmol; PerkinElmer) (~250 Bq), varying amounts of standard E_2_, and a constant amount of each scFv (adjusted to bind to ~50% of the tritium-labeled E_2_) were incubated in G-PBS (500 μL) at 4 °C for 240 min. The bound (B) and free (F) fractions were separated using a dextran-coated charcoal method, and the radioactivity of the B fraction was measured.
- **SPR analysis**. The kinetic parameters of selected scFvs, which were affinity-purified with anti-FLAG-M2 agarose (Sigma–Aldrich)^13^, to the E_2_–bovine serum albumin (BSA) conjugate (prepared according to the previous method^12^: E_2_/BSA molar ratio was determined to be 7) immobilized on the CM5 sensor chip (using an Amine Coupling) were determined with Biacore T200 SPR system (GE Healthcare). Kinetic measurements were carried out at 25 °C in G-PBS with a constant flow rate of 30 μL/min. In the measurements, five different concentrations (0.16–10 μg/mL) of the purified scFvs were used. The kinetic evaluation of data was performed using Biacore T200 evaluation software (GE Healthcare).

### ELISA

The 96-well microplates (#3590; Corning) coated with the E_2_–BSA conjugate (see above) were incubated at 4 °C for 240 min with a mixture of E_2_ standard (50.0 μL/well) and soluble scFv protein (100 μL/well), both diluted in PVG-PBS. The microplates were washed 3 times with T-PBS and probed with an anti-FLAG M2 antibody labeled with peroxidase (POD) (Sigma–Aldrich) diluted in G-PBS (0.20 μg/mL; 100 μL/well)^12–17^. After incubation at 37 °C for 30 min, the microplates were washed similarly and the captured POD activity was determined colorimetrically (490 nm), as described previously^12–17^. To construct the ELISA dose–response curves, Image J software^48^ (NIH) was used for curve fitting and determining the reaction parameters. The midpoint (*i.e*., IC_50_) values were derived from a four parametric logistic equation [log(analyte dose) vs. B/B_0_(%)] as the EC50 values. The unit “X g/assay” was used in the abscissa, which refers to the total mass (X g) of analyte that was added to each assay chamber (microwell) for the competitive antigen–antibody reactions.

## Supplementary information

Supplementary information.

## Data Availability

The data sets generated during the current study are available from the corresponding author upon request.
