# Supplementary material for: Seeking high-priority mutations enabling successful antibody-breeding: systematic analysis of a mutant that gained over 100-fold enhanced affinity
Source: Sci Rep. 2020 Mar 16;10:4807. doi: 10.1038/s41598-020-61529-7 (PMC7075871; doi:10.1038/s41598-020-61529-7)
Supplement: Supplementary file 1 — Supplementary information. [file 41598_2020_61529_MOESM1_ESM.pdf]

Supplementary information

Seeking high-priority mutations enabling successful antibody-breeding: systematic analysis of a mutant that gained over 100-fold enhanced affinity

Hiroyuki Oyama, Yuki Kiguchi, Izumi Morita, Chika Yamamoto, Yuka Higashi, Miku Taguchi, Tatsuya Tagawa, Yuri Enami, Yuriko Takamine, Hanako Hasegawa, Atsuko Takeuchi, and Norihiro Kobayashi*

Kobe Pharmaceutical University, 4-19-1, Motoyama-Kitamachi, Higashinada-ku, Kobe 658-8558, Japan

Supplementary figures and table

(A)

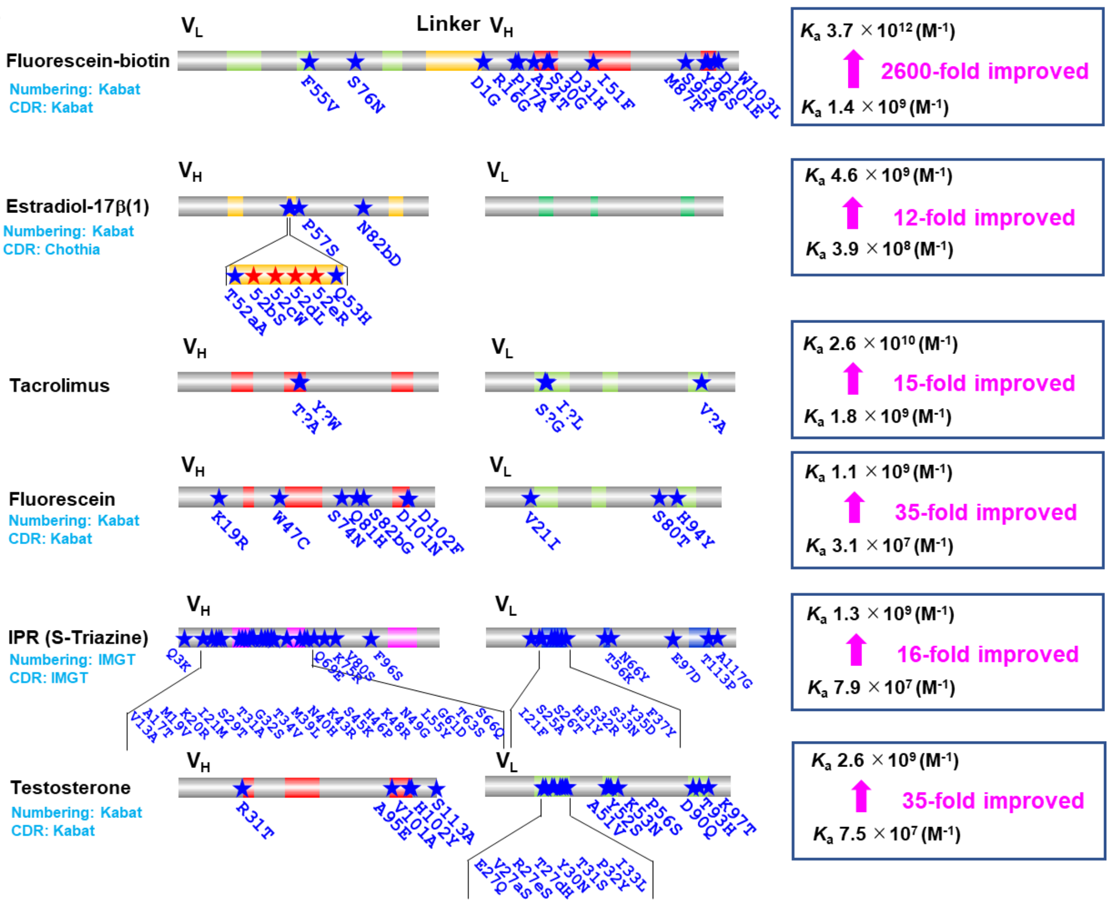

(B)

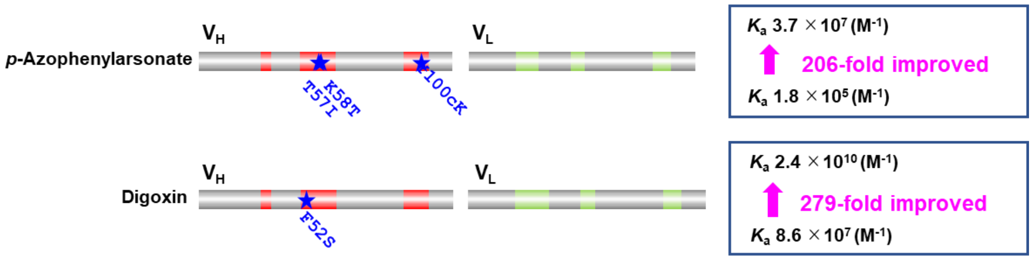

Figure S1. (A) Schematic illustration of the primary structures of successfully affinity-matured antibody fragments (scFv or Fab) targeting small molecules, each with a K_a value $>10^9$ M^{-1}, which were generated from the corresponding parent antibody with a >10-fold improvement in the K_a (but the affinity-matured scFvs we produced, referred to in the text, are not cited). Amino acid substitutions observed are indicated with blue stars and one-letter codes. The amino acids introduced by site-directed insertions are shown with red stars. Each K_a value and the magnitude of the improvement are also shown in the boxes. The associated references are as follows: estradiol-17β(1)[1], fluorescein–biotin[2], IPR (*S*-triazine derivative)[3], testosterone[4], fluorescein[5], and tacrolimus[6]. The findings of these studies were also described in detail in our previous articles[7,8]. (B) The primary structures of the V_H and V_L domains of hybridoma-derived antibodies against *p*-azophenylarsonate[9] and digoxin[10] are also shown: these structures are those of antibody species showing higher affinity among their closely related antibodies. The amino acid substitutions (as above) and improvements of K_a values (indicate the difference between the higher-affinity and lower-affinity antibodies) are shown in the boxes.

The CDRs in the V_H domain (H1→H2→H3) and V_L domain (L1→L2→L3) are shown in red and pale green, respectively. It should be noted that these studies employed different CDR definitions: *i.e.*, Chothia et al.[11] (ref. 1), Kabat et al.[12] (ref. 2, 4, and 6), IMGT[13] (ref. 3), or a modified definition by the authors of this study (ref. 6).

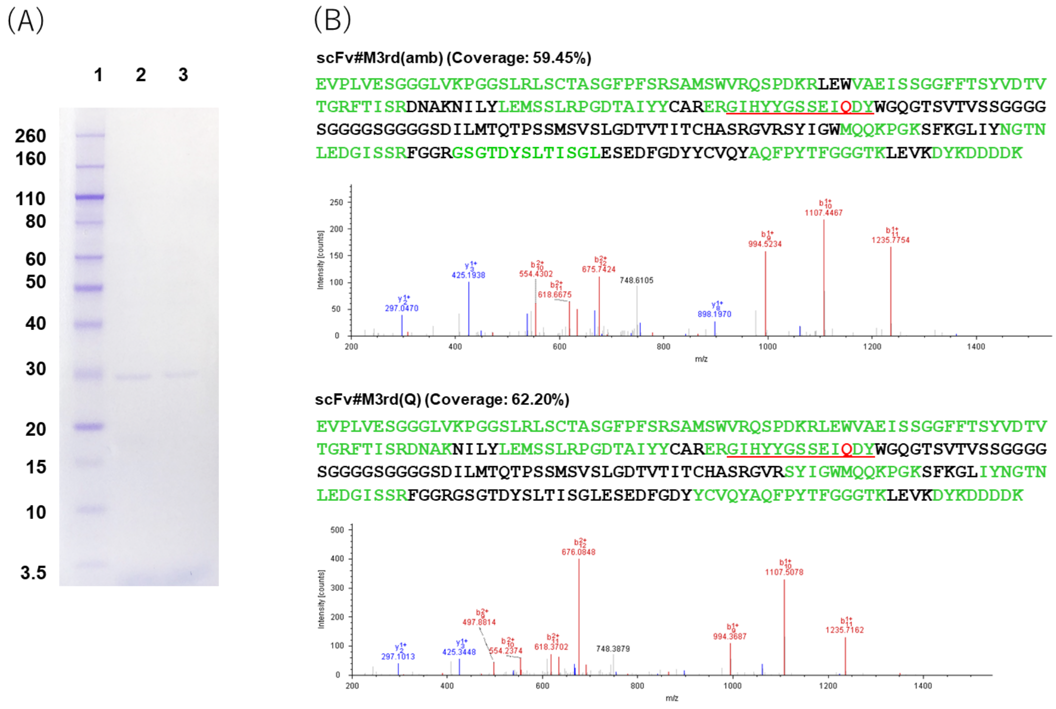

Figure S2. Determining the identity of the scFv#M3rd(amb) and scFv#M3rd(Q) proteins.

(A) SDS-PAGE with Coomassie brilliant blue staining of the affinity-purified soluble scFv proteins. Lane 1, M_r marker; lane 2, scFv#M3rd(amb); lane 3, scFv#M3rd(Q).

(B) Summary of the LC/MS/MS fingerprinting analysis. Partial sequences identified by LC/MS/MS are indicated with green letters and placed in the context of the entire scFv sequence, and the coverage (%) is shown. The MS/MS spectra, assigned to the tridecapeptide GIHYYGSSEIQDY (underlined) derived from scFv#M3rd(amb) and scFv#M3rd(Q) (underlined), are shown. We observed b_{11}^{+} ions [calcd for GIHYYGSSEIQ, *m/z* 1235.56915; found 1235.7754 for scFv#M3rd(amb) and 1235.7162 for scFv#M3rd(Q)], b_{11}^{2+} ions [calcd for GIHYYGSSEIQ, *m/z* 618.28821; found 618.6675 for scFv#M3rd(amb) and 618.3702 for scFv#M3rd(Q)], and y_3^{+}-ion [calcd for QDY, *m/z* 425.16669; found 425.1938 for scFv#M3rd(amb) and 425.3448 for scFv#M3rd(Q)], which indicate the presence of Q at the V_H100g position (shown in red). **Methods:** The scFv proteins were run on a polyacrylamide gel, and each gel slice containing the protein band was suspended in Tris-HCl buffer (~100 μg/mL) and digested with trypsin using XL-Tryp kit (Apro Science). LC/MS/MS was performed on an LTQ-Orbitrap Discovery (linear ion trap–orbitrap) spectrometer (Thermo Fisher Scientific). The mobile phases consisted of 0.10% formic acid in water (solvent A) and 0.10% formic acid in acetonitrile (solvent B). A solution of the scFv-derived peptides, dissolved in 0.10% trifluoroacetic acid (35 μL), was applied to the LC/MS/MS system. The peptides were fractionated on an L-column Micro C-18 (150 mm length × 0.10 mm diameter; particle size, 3 μm; Chemicals Evaluation and Research Institute) with a linear gradient of 3.0–43% solvent B for 40 min at a flow rate of 500 nL/min. The column eluent was sprayed directly into the ion source of the mass spectrometer. The mass spectra were measured over a range of 300–2000 *m/z* ratio. In the mass spectra for both eluents, the top seven high-intensity precursor ions were

selected automatically for subsequent product ion analysis. The LC/MS/MS data were interpreted using Proteome Discoverer (Thermo Fisher Scientific). Peptides were identified from the self-made database, with a peptide mass tolerance of 4 ppm and a fragment mass tolerance of 0.80 Da.

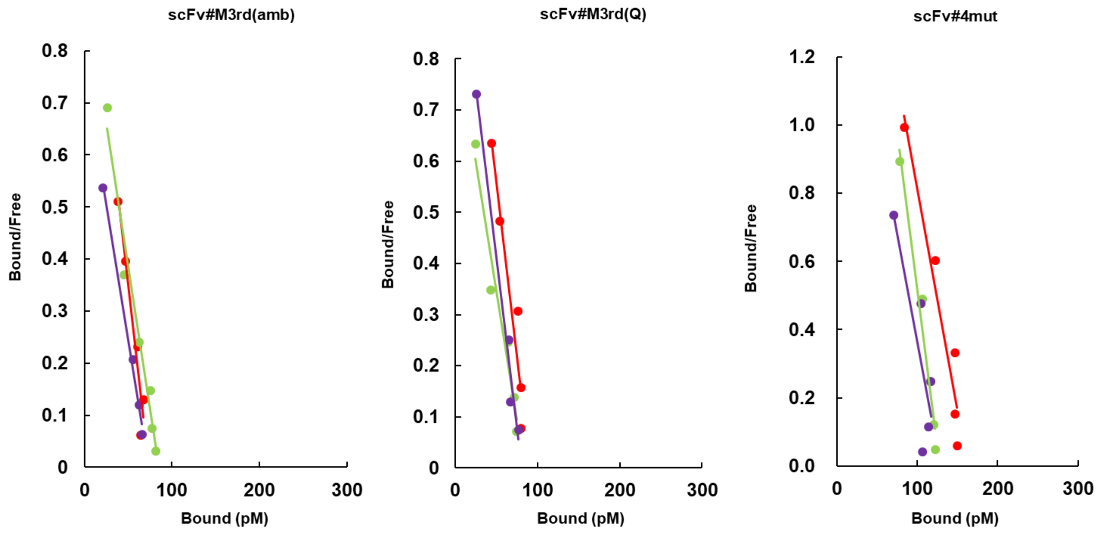

Figure S3. The results of Scatchard analysis[14] for scFv#M3rd(amb), scFv#M3rd(Q), and scFv#4mut are shown. The dark red, pale green, and purple lines mean three determinations performed on each scFv.

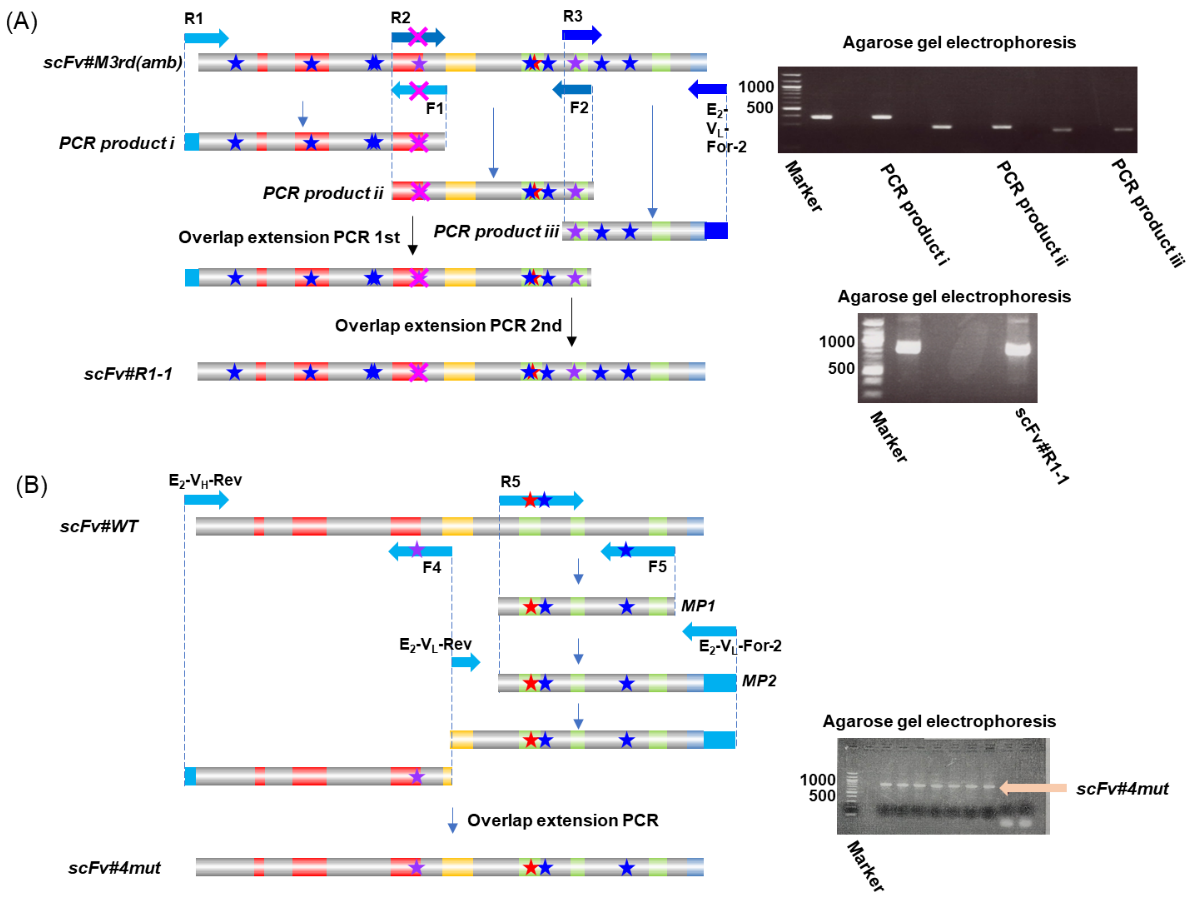

Figure S4. Schematic illustrations of the strategies used to construct gene fragments encoding (A) scFv#R1-1 and (B) scFv#4mut. Gel electrophoresis analysis of the PCR products was performed on a 2% agarose gel run in standard Tris/acetic acid/ethylenediaminetetraacetic acid buffer at 100 V.

Table S1. Nucleotide sequences of PCR primers used in this study.

Primer	Sequences (5′→3′) showing restriction sites[a]
R1	ATTGTTATTACTCGCGGCCCAACCGGCCATGGCCGA
R2	TACGGAAGTAGCGAAATC**TTG**GACTACTGGGGTCAAGGA
R3	TTTAAGGGCCTGATCTATAATGGAACCAACTTGGAAGAT
R4	TACGGAAGTAGCGAAATTGAGGACTACTGGGGTCAAGGA
R5	CATGCAAGTCAGGGC**GTT**AGAAGTTATATCGGGTGG**ATG**CAGCAGAAACCA
F1	TCCTTGACCCCAGTAGTC**CAA**GATTTCGCTACTTCCGTA
F2	ATCTTCCAAGTTGGTTCCATTATAGATCAGGCCCTTAAA
F3	AGAGGCTGTACAGGAGAG**TTT**TAGGGATCCTCCAGGCTT
F4	CCGCCGGATCCACCTCCGCCTGAACCGCCTCCACCTGAGGAGACGGTGACTGAGGTTCC TTGACCCCAGTAGTC**CTG**GATTTC
F5	ATCTTCAGATTCCAG**GCC**GCTGATGGTAAGAGA
E_2-V_H-Rev	ATTGTTATTACTCGCGGCCCAACCGGCCATGGCCGAAGTGCCACTGGTGGAGTCTGGG
E_2-V_H-For	CCGCCGGATCCACCTCCGCCTGAACCGCCTCCACCTGAGGAGACGGTGACTGAGGTTCC
E_2-V_L-Rev	CAGGCGGAGGTGGATCCGGCGGTGGCGGATCGGATATTTTGATGACCCAAACTC
E_2-V_L-For-2	GATTTGGGCTCAACTTTCTTGTCGACTTATTATTTATCATCATCATCTTTATAATCT

[a] Restriction sites are underlined, and the codons for introducing missense mutations are shown with bold letters.

References

1. Lamminmäki, U., *et al*. Expanding the conformational diversity by random insertions to CDRH2 results in improved anti-estradiol antibodies. *J. Mol. Biol.* **291**, 589–602 (1999).
2. Boder, E. T., Midelfort, K. S. & Wittrup, K. D. Directed evolution of antibody fragments with monovalent femtomolar antigen-binding affinity. *Proc. Natl. Acad. Sci. U. S. A.* **97**, 10701–10705 (2000).
3. Kramer, K. Evolutionary affinity and selectivity optimization of a pesticide-selective antibody utilizing a hapten-selective immunoglobulin repertoire. *Environ. Sci. Technol.* **36**,

4892–4898 (2002).
4. Valjakka, J. *et al*. Crystal structure of an *in vitro* affinity- and specificity-matured anti-testosterone Fab in complex with testosterone. *J. Biol. Chem.* **277**, 44021–44027 (2002).
5. Fukuda, I. *et al. In vitro* evolution of single-chain antibodies using mRNA display. *Nucleic Acids Res.* **34**, e127 (2006).
6. Siegel, R. W., Baugher, W., Rahn, T., Drengler, S. & Tyner, J. Affinity maturation of tacrolimus antibody for improved immunoassay performance. *Clin. Chem.* **54**, 1008–1017 (2008).
7. Kobayashi, N. & Oyama, H. Antibody engineering toward high-sensitivity high-throughput immunosensing of small molecules. *Analyst* **136**, 642–651 (2011).
8. Oyama, H., Yamaguchi, S., Nakata, S., Niwa, T. & Kobayashi, N. "Breeding" diagnostic antibodies for higher assay performance: a 250-fold affinity-matured antibody mutant targeting a small biomarker. *Anal. Chem.* **85**, 4930–4937 (2013).
9. Sharon, J. Structural correlates of high antibody affinity: three engineered amino acid substitutions can increase the affinity of an anti-*p*-azophenylarsonate antibody 200-fold. *Proc. Natl. Acad. Sci. U.S.A.* **87**, 4814–4817 (1990).
10. Schildbach, J. F. *et al*. Altered hapten recognition by two anti-digoxin hybridoma variants due to variable region point mutations. *J. Biol. Chem.* **266**, 4640–4647 (1991).
11. Chothia, C. *et al*. Conformations of immunoglobulin hypervariable regions. *Nature* **342**, 877–883 (1989).
12. Kabat, E. A., Wu, T. T., Perry, H. M., Gottesman, K. S. & Foeller, C. *Sequences of Proteins of Immunological Interest*. U.S. Department of Health and Human Services, National Institutes of Health (U.S. Government Printing Office: Washington, DC), (1991).
13. Welcome! To the IMGT home page. http://www.imgt.org/
14. Scatchard, G. The attractions of proteins for small molecules and ions. *Ann. N. Y. Acad. Sci.* **51**, 660–672 (1949).
